# Factors explaining the heterogeneity of effects of patient decision aids on knowledge of outcome probabilities: a systematic review sub-analysis

**DOI:** 10.1186/2046-4053-2-95

**Published:** 2013-10-21

**Authors:** Stephen J Gentles, Dawn Stacey, Carol Bennett, Mohamad Alshurafa, Stephen D Walter

**Affiliations:** 1 Department of Clinical Epidemiology and Biostatistics, McMaster University, Hamilton, ON, Canada; 2 School of Nursing, University of Ottawa, Ottawa, ON, Canada; 3 Ottawa Hospital Research Institute, Ottawa, ON, Canada; 4 Health Information Research Unit, McMaster University, 1280 Main Street West, CRL 126, Hamilton, ON L8S 4K1, Canada

**Keywords:** Decision aid, Clinical heterogeneity, Meta-analysis, Meta-regression, Subgroup analysis, Effect modification, Baseline rate, Control event rate

## Abstract

**Background:**

There is considerable unexplained heterogeneity in previous meta-analyses of randomized controlled trials (RCTs) evaluating the effects of patient decision aids on the accuracy of knowledge of outcome probabilities. The purpose of this review was to explore possible effect modification by three covariates: the type of control intervention, decision aid quality and patients' baseline knowledge of probabilities.

**Methods:**

A sub-analysis of studies previously identified in the 2011 Cochrane review on decision aids for people facing treatment and screening decisions was conducted. Additional unpublished data were requested from relevant study authors to maximize the number of eligible studies. RCTs (to 2009) comparing decision aids with standardized probability information to control interventions (lacking such information) and assessing the accuracy of patient knowledge of outcome probabilities were included. The proportions of patients with accurate knowledge of outcome probabilities in each group were converted into relative effect measures. Intervention quality was assessed using the International Patient Decision Aid Standards instrument (IPDASi) probabilities domain.

**Results:**

A main effects analysis of 17 eligible studies confirmed that decision aids significantly improve the accuracy of patient knowledge of outcome probabilities (relative risk = 1.80 [1.51, 2.16]), with considerable heterogeneity (87%). The type of control did not modify effects. Meta-regression suggested that the IPDASi probabilities domain score (reflecting decision aid quality) is a potential effect modifier (*P* = 0.037), accounting for a quarter of the variability (*R*^2^ = 0.28). Meta-regression indicated the control event rate (reflecting baseline knowledge) is a significant effect modifier (*P* = 0.001), with over half the variability in ln(OR) explained by the linear relationship with log-odds for the control group (*R*^2^ = 0.52); this relationship was slightly strengthened after correcting for the statistical dependence of the effect measure on the control event rate.

**Conclusions:**

Patients’ baseline level of knowledge of outcome probabilities is an important variable that explains the heterogeneity of effects of decision aids on improving accuracy of this knowledge. Greater relative effects are observed when the baseline proportion of patients with accurate knowledge is lower. This may indicate that decision aids are more effective in populations with lower knowledge.

## Background

In systematic reviews of binary outcomes, heterogeneity conventionally refers to the variation in relative effects (relative risk, odds ratio) across studies that is greater than one would expect by chance [1]. The causes of such study-level variation can either be artifactual, where methodological differences between studies affect the relative effect measures, or real, where differences may be attributable to variation across studies in factors related to the population included, active interventions used or comparators employed [2,3]. When present, unexplained heterogeneity complicates the interpretation and usefulness of pooled effect estimates of meta-analyses in decision-making. It is for this reason that the quality of pooled evidence is typically downgraded when assessed using the GRADE framework [4]. Attempts to explain sources of heterogeneity are important for overcoming these limitations and for their potential to contribute knowledge about what types of patients benefit most from a specific intervention [2,3,5,6]. The Cochrane meta-analysis of randomized controlled trials (RCTs) evaluating patient decision aid effects on the accuracy of knowledge of outcome probabilities is an example where interpretation of the pooled effect has been hampered by high heterogeneity.

Patient decision aids are complex interventions used to help patients make specific and deliberative choices among treatment or screening options by providing, at the minimum, information on the options and associated outcomes relevant to the patient’s health status, and implicit methods to clarify their values or preferences [7]. Due to their complex nature – involving multiple interacting components and behaviors – and the diverse clinical settings they are designed for, the exact form of the intervention and populations in which they are evaluated vary considerably. There is thus a corresponding expectation of variation in real decision aid effects across conditions.

The effects of decision aids on numerous decision-related outcomes have been extensively evaluated. Since patients are known to underestimate probabilities of harms or overestimate probabilities of benefits [8], decision aids are often designed to communicate estimates of probabilities derived from population-based research. Such probabilities apply to possible outcomes of the featured decisions: benefits and harms of an intervention, or true- and false-positive or -negative screening results [9]. Studies that evaluate the effects of decision aids on the accuracy of patient knowledge of these outcome probabilities generally measure the proportion of patients who are able to correctly answer questions about population-derived probability estimations – making this a binary outcome.

The most recent (2011) update to the Cochrane systematic review on patient decision aids includes 86 RCTs where the authors reviewed 23 different outcomes [7]. Accuracy of knowledge of outcome probabilities (labeled ‘accurate risk perception’ in that review) was the second-most frequently measured outcome, and the results of 14 studies were pooled. Meta-analysis revealed a uniform direction of effect favoring decision aids across all studies, and the pooled effect estimate was significant (relative risk = 1.74 [1.46 to 2.08], *P* < 0.001). The level of heterogeneity, however, was significant (*P* < 0.001) and considerable (*I*^2^ = 83%). Despite this, the pooled effect is considered informative to a degree since decision aids showed a uniformly positive effect. However, the Cochrane review mentions that ‘the pooled effect size and CI should be interpreted as a range across conditions, which may not be applicable to a specific condition’ [10], reflecting the limitation to the interpretability and utility of the pooled random effects estimates found in meta-analyses when there exists substantial real variation in intervention effects. In other words, the pooled estimate does not correspond to any individual decision aid, setting or population. Furthermore, it is impossible to predict where any given decision aid would lie within the wide range of possible relative effects [3].

The 2011 Cochrane update [7] tentatively explored two sources of heterogeneity affecting this outcome. First, it showed that the effect size of decision aids in which probabilities were represented numerically is larger than for those where probabilities were described with words, suggesting possible effect modification attributable to this specific aspect of the intervention. Secondly, removing three (of 14) studies with the lowest control event rate (selected as outliers by visual inspection) reduced heterogeneity to non-significant levels (*P* = 0.3), implicating control levels of accurate knowledge as a potential contributor to heterogeneity [10]. While informative, these preliminary analyses were not selected with any overall rationale and did not provide formal tests for effect modification.

The current investigation aims to improve interpretability and usefulness of the available research evidence regarding decisions aid effects on the accuracy of patient knowledge of outcome probabilities by exploring and characterizing potential contributors to the observed heterogeneity [2-4]. Subgroup analysis and meta-regression were employed to investigate the potential effects of three study-level factors (covariates): the type of control intervention, the level of decision aid quality and the control event rate. These covariates were chosen because they represent the best available measures that summarize or combine relevant characteristics of the comparator (control), active intervention or study population, respectively.

## Methods

As a sub-analysis of the previous Cochrane systematic review on decision aids [7], certain aspects of the original methods were not repeated in detail here – principally the literature search and parts of the literature selection. In addition, the original review can be consulted for further information on individual studies, including setting, patients included, intervention characteristics and risk of bias assessments.

### Data sources and study selection

Studies previously identified through electronic database searches (MEDLINE, PsycINFO, CINAHL, EMBASE, Cochrane Central Controlled Trials Register) in the 2011 Cochrane review served as the basis for study selection [7]. Thus, RCTs published up to December 2009 meeting the original selection criteria were considered. As an additional criterion, we included studies where data had been collected on the proportions of participants in both intervention and control groups who had accurate knowledge of outcome probabilities post-intervention. To maximize the number of studies available for analysis, the 86 publications included in the 2011 Cochrane update were rescreened to identify studies where the relevant outcome data might exist but had not been previously published. The corresponding authors were then emailed up to three times requesting unpublished data used to calculate relative risks and copies of the original decision aids.

### Data extraction

Data from all studies were extracted in duplicate (SG, MA) using piloted forms. In addition to newly eligible studies, data were re-extracted from the set of 14 studies pooled in the 2011 Cochrane update [7]. In cases of disagreement with the outcome data from the previous Cochrane review, its authors (CB, DS) were consulted and consensus was reached on which data to use for the current review.

Event rates, defined as the proportion of patients in the decision aid group correctly answering questions about probabilities divided by that in the control group, were extracted for calculating relative risk. In eight studies that evaluated knowledge of outcome probabilities with more than one question, the proportion of correct answers was averaged. For purposes of GRADE assessment, the risk of bias items applicable at the outcome level (blinding, incomplete outcome data, specifically for assessments of knowledge of probabilities) were abstracted, as these items were previously reported in the Cochrane update [7] only at the study level. Information for the three covariates analyzed was abstracted (described below).

### Selection of study-level factors (covariates) investigated

Study-level factors with the potential to contribute to heterogeneity (covariates) were considered to represent three principal sources of clinical heterogeneity: characteristics of the comparator (control), the active intervention and the population [2]. To minimize the risk of detecting spurious effect modification due to multiple comparisons, only one covariate was selected to represent each main source, to give a total of three [11,12]. In each case, the covariates were selected for their availability and biologic plausibility (likelihood based on a mechanistic rationale) as substantial contributors to heterogeneity [11]. For the first category, comparator (or control), only one covariate was available and therefore selected: the type of control intervention. Since multiple covariates were available corresponding to characteristics of active intervention and study population, a top-down approach was used in which the best available measure that combined potentially relevant characteristics was selected in each case. To represent intervention characteristics, a composite measure of relevant decision aid quality characteristics was chosen. For population characteristics, the control event rate was chosen because it provides a convenient summary measure [13]. The rationale, hypothesis and measurement for each covariate are described below.

#### Type of control intervention

Depending on the context, not all studies evaluating decision aids provide the same degree of standardized information to the control group [7]. Three types of control intervention, from less to more standardized information, are categorized: (1) no standardized information other than usual care; (2) generic standardized information used as a sham, such as basic background on the disease, and containing no outcome information or (3) information on outcomes associated with options, sometimes considered as a less intense form of decision aid. In all cases, control interventions differ from the experimental intervention by providing no information on outcome probabilities. Higher levels of standardized information may have a hidden effect on patients’ ability to answer questions about probabilities. The hypothesis for this covariate was that control interventions that provide more standardized information to the control group, because they may conceivably improve control patients’ ability to answer questions about probabilities, would decrease relative effect size. Possible effect modification by this categorical covariate was investigated with subgroup analysis.

#### Decision aid quality

The International Patient Decision Aid Standards (IPDAS) collaboration has developed an instrument, the IPDASi, for rating the quality of decision aids [14]. IPDASi includes a probabilities dimension consisting of eight items corresponding to theoretical elements derived from systematic review of the evidence on effective formats for communicating outcome probabilities to patients [15]. The items address factors including the presentation of event rates, specification of a time period, the allowing for comparison of probabilities across options, the reporting of levels of uncertainty around probabilities, the provision of multiple ways of viewing probabilities (for example, words, numbers and diagrams) and providing balanced information to limit framing biases [14]. The probabilities dimension therefore represents a comprehensive composite measure of relevant decision aid characteristics likely to affect knowledge of probabilities. Moreover, its continuous scale probably gives greater statistical power when testing for effect modification than does an equivalent categorical variable. The hypothesis for this covariate was that decision aid scores on the IPDASi probabilities dimension would increase as the effectiveness of decision aids for improving knowledge of outcome probabilities increases – which, if true, would support the predictive validity of the probabilities dimension of IPDASi [14]. Decision aids were scored in duplicate by trained raters on a scale from 1 to 4 points for each of 8 items in this dimension (scores provided by NJW). The possible ratings of 8 to 32 were re-scaled to a range of 0% to 100%. The effects of this continuous covariate were investigated with meta-regression.

#### Control event rate

The control event rate (CER) in this context is the proportion of patients in the control group who correctly answer specific questions about probabilities. Note, ‘control event rate’ is used in preference to ‘baseline risk’ to minimize confusion, since ‘risk’ in this case corresponds to a favored outcome (that is, having accurate knowledge of probabilities). Assuming the type of control intervention does not modify its effects (and our investigations found no evidence that it does), the control event rate provides an estimate of the baseline level of accurate knowledge of outcome probabilities in the population studied. Patients’ baseline knowledge of these probabilities may vary widely depending on factors such as whether specific probabilities are likely to be common knowledge, newness of the underlying evidence or patient education levels. The plausibility of effect modification was first suggested in the 2009 Cochrane update where heterogeneity was reduced to non-significant levels after removing three studies with the lowest control event rate [10]. The hypothesis for this covariate was that studies with higher control event rates have lower relative risks. Effects due to this continuous covariate were investigated with meta-regression.

### Analysis

Three types of statistical analysis were performed: meta-analysis of main effects, subgroup analysis to test for effect modification by the one categorical covariate (type of control intervention) and meta-regression to test for and characterize effect modification by the two continuous covariates (decision aid quality and control event rate). Each analysis type is described in further detail. The threshold for statistical significance was *P* < 0.05.

#### Meta-analysis of main effects

Consistent with previous meta-analysis of the main effects for this outcome [7], relative risk was used as the effect measure. The software Review Manager (RevMan, version 5.1, Copenhagen, The Nordic Cochrane Centre, The Cochrane Collaboration, 2011) was used to combine estimates using the DerSimonian and Laird random-effects model. Tau-squared in this model provides an estimate of the between-study variance. A chi-squared test was used to examine the strength of evidence about whether heterogeneity is present, and *I*^2^ provides an estimate of its magnitude.

#### Subgroup analysis (type of control intervention)

Potential effect modification by the three types of control intervention was tested with a weighted one-way ANOVA. To provide additional support for a lack of effect on the control event rate, a weighted ANOVA between type of control intervention and control event rate was performed. ANOVAs were calculated using the software IBM SPSS Statistics (version 20.0 for Windows, Armonk, NY, IBM Corp.), using the natural logarithm of the odds ratio, ln(OR), as the effect measure for consistency with subsequent covariate analyses.

#### Meta-regression analysis (decision aid quality and control event rate)

Univariate weighted least squares (WLS) meta-regression analyses were conducted to test for and characterize potential effect modification by IPDASi probabilities dimension score and control event rate, separately.

In selecting the most appropriate scales for these analyses, the effect measure was first considered. Changing the effect measure (between relative risk (RR), OR, or ln(OR)) and scale for representing the relationship has been recommended as a strategy to minimize apparent heterogeneity and effect modification as a first step in reducing the chance of detecting a spurious interaction in meta-regression where control event rate is a covariate [6,16,17]. Of the three effect measures, ln(OR) had the lowest heterogeneity (*I*^2^, Table 1) and was found in exploratory analyses to have the least significant slope vs control event rate, providing justification for using this effect measure in the meta-regression. As additional justification, the natural log of the OR is commonly chosen because it has better statistical properties since zero is the value of no effect [6]. For the analysis of decision aid quality, ln(OR) was plotted against the re-scaled IPDASi probabilities dimension score (0% to 100%). For the analysis of control event rate, ln(OR) was plotted against log-transformed values of the control event rate (that is, logit control) so that both variables could share the same scale making a linear model easier to interpret. With ln(OR) as the common effect measure, exploratory multiple regression combining the CER and IPDASi probabilities dimension score could be performed more easily.

**Table 1:** Study-level covariate values, observed effect size measures and pooled heterogeneity estimates listed in order of increasing relative risk

**Study**	**Type of control**^**a**^	**IPDASi, probability dimension score/32**	**Rescaled IPDASi, probability dimension score (%)**	**CER**	**Logit control**	**ln(OR)**	**OR**^**b**^	**RR**^**b**^
Lerman *et al.*[28]	A	16	33	0.66	0.65	0.37	1.46	1.12
Johnson *et al*. [32]	A	16	33	0.77	1.17	0.96	2.86	1.17
Wolf and Schorling [24]	B	19	46	0.54	0.16	0.73	2.08	1.31
Whelan *et al*. [25]	A	17	38	0.58	0.32	0.91	2.52	1.34
McBride *et al*. [27]	A	23	63	0.30	−0.85	0.49	1.64	1.37
Schapira and Vanruiswyk [23]	B	28	83	0.47	−0.10	0.89	2.45	1.45
Dodin *et al*. [31]	B	26^c^	75	0.43	−0.28	0.82	2.32	1.48
O’Connor *et al*. [8]	C	26^c^	75	0.46	−0.14	1.05	2.91	1.54
Whelan *et al*. [21]	B	22	58	0.37	−0.53	0.82	2.29	1.55
Kuppermann *et al*. [36]	C	NA	NA	0.32	−0.76	1.35	3.88	2.03
Vandemheen *et al*. [37]	B	30	92	0.29	−0.88	1.52	4.67	2.26
McAlister *et al*. [29]	A	29^d^	88	0.16	−1.62	1.11	3.06	2.29
Mathieu *et al*. [34]	B	31	96	0.22	−1.29	1.54	4.71	2.62
Man-Son-Hing *et al*., [30]	A	29^d^	88	0.24	−1.16	1.83	6.32	2.80
Weymiller *et al*. [35]	B	32	100	0.18	−1.48	1.88	6.94	3.38
Laupacis *et al*. [33]	A	24	67	0.08	−2.34	1.50	4.88	3.72
Gattellari and Ward [22]	B	21	54	0.10	−2.14	2.29	10.26	5.28
**Chi-squared (heterogeneity)**						55.75	56.41	120.19
**I**^**2**^						71%	72%	87%

a A, no standardized information; B, standardized generic information (no outcome information); C, simple decision aid (no standardized probability information).

b No continuity correction was applied to match Review Manager’s output.

c Same decision aid for both trials.

d Same decision aid for both trials.

CER, control event rate; IPDASi, International Patient Decision Aid Standards instrument; NA, full decision aid not available for rating; OR, odds ratio; RR, relative risk.

Since the selected effect measure ln(OR) is not available in RevMan, Excel was used to generate an equivalent meta-analysis for ln(OR) to obtain the tau-based weights for the meta-regressions. Excel formulae were verified by comparing (non-continuity-corrected) back-translated values to the RevMan output for OR. Event frequencies for this meta-analysis were continuity-corrected (adding 0.5). IBM SPSS Statistics was then used to calculate standard WLS regressions using the tau-based weights. Neither regression model (logit control vs ln(OR) or re-scaled IPDAS vs ln(OR)) was found to violate the assumptions of linear regression (linearity, independence, homoscedasticity and normality) upon examination of the residual plots (predicted vs residual, independent vs residual and normal probability (Q-Q) of residual plots).

The meta-regression against control event rate incorporated a bias correction. When baseline response rates (control event rates) are used as the covariate in a meta-regression, the measurement error in control event rate and the functional dependence of the observed treatment effect on the control group response can bias the standard WLS regression and lead to incorrect inference about the degree to which the control event rate modifies effects and underlies heterogeneity [13,16,18]. This problem was addressed using a modified WLS approach developed previously [18], which considers sampling error in the control event rate and generates bias terms that are used to correct the standard regression coefficients. Bias terms and bias-corrected regression coefficients were calculated using Excel, the formulae for which were verified using data from the original article describing this approach [18].

To calculate relative risk values predicted by the bias-corrected regression formula for corresponding control event rate values, back-translation was performed using Excel.

#### GRADE assessment

The GRADE framework was employed to provide a standardized summary rating of the pooled evidence for the outcome of interest based on key quality dimensions: risk of bias, consistency, directness, precision and publication bias [4,19,20]. The software GRADEpro (version 3.2 for Windows, 2008) was used.

## Results

### Meta-analysis of main effects

**Figure 1:**
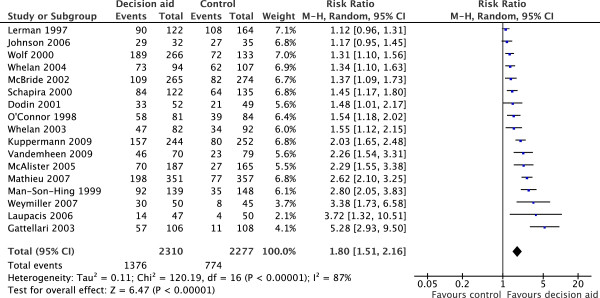
**Main effects of decision aids on patient knowledge of outcome probabilities.** CI, confidence interval; df, degrees of freedom; RR, relative risk.

Of 86 studies from the 2011 Cochrane review, 17 studies were included in the current meta-analysis of the effects of decision aids on the accuracy of knowledge of outcome probabilities [8,21-37]. Efforts to obtain additional unpublished data resulted in three studies [32,34,35] being added to the 14 from the 2011 Cochrane analysis for this outcome. The authors of three additional studies who were contacted either confirmed that relevant data was unavailable (*n* = 1) or were unable to provide data (*n* = 2). Figure 1 shows the main pooled relative effect for the outcome accuracy of patients’ knowledge of outcome probabilities was significant, with a uniform direction of effect favoring decision aids (relative risk = 1.80 [1.51, 2.16]); heterogeneity was significant (*P* < 0.001) and considerable (*I*^2^ = 87%).

### Subgroup and meta-regression analysis of covariate effects

Table 1 shows the covariate values and corresponding effect sizes for each study. For the subgroup analysis that tested effect modification due to the type of control intervention used (no standardized information, generic information or simple decision aid without probability information), the weighted ANOVA was not significant (*F* = 2.33, degrees of freedom, df = 2, *P* = 0.11). As further support for the lack of effect of the type of control intervention on the control event rate, the second ANOVA between these two covariates also lacked significance (*F* = 0.49, df = 2, *P* = 0.62).

Table 2 summarizes the relationships corresponding to each of the two meta-regression analyses: decision aid quality (rescaled IPDASi probabilities dimension score) vs effect size, and log-transformed control event rate vs effect size before and after bias correction.

**Table 2:** Regression coefficients for normalized IPDASi probabilities dimension score vs ln(OR) and logit control vs ln(OR)

		**Intercept**	**Slope (standard errorcpa**
(a)	Normalized IPDASi probabilities score vs ln(OR)	0.25	0.013 (0.006)
(b)	logit control vs ln(OR), non-bias-corrected	0.86	−0.436 (0.108)
	logit control vs ln(OR), bias-corrected	0.88	−0.466

IPDASi, International Patient Decision Aid Standards instrument; OR, odds ratio.

**Figure 2:**
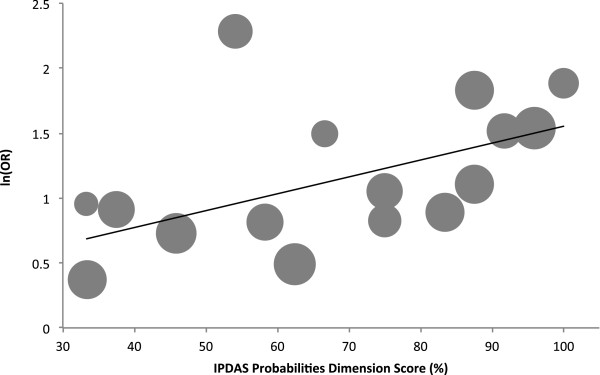
**Meta-regression of the effect of decision aid quality: normalized IPDASi probabilities dimension score vs ln(OR).** Kuppermann *et al*. [36] is excluded since this decision aid was not available for scoring on the IPDASi probabilities dimension. The area of each circle is proportional to the weight for that study. IPDASi, International Patient Decision Aid Standards instrument; OR, odds ratio.

The quality (IPDASi probabilities dimension scores) of the decision aids evaluated in the included studies ranged widely from 16 to 32 out of a total possible score of 32 (33.3% to 100% when rescaled). The slope of the univariate regression relationship between the rescaled quality scores (%) and ln(OR) (Figure 2) was significant (intercept 0.253, slope 0.013, *P* = 0.037), and accounted for a quarter of the variability in effect size between studies (*R*^2^ = 0.28).

**Figure 3:**
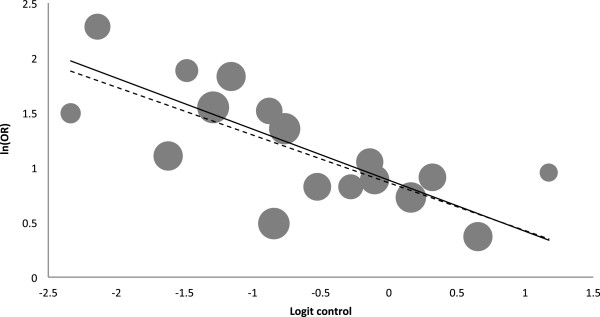
**Meta-regression of the effect of control event rate: logit control vs ln(OR).** The dashed line is prior to bias correction. The solid line is after bias correction. The area of each circle is proportional to the weight for that study. OR, odds ratio.

The control event rate (representing the proportion of control patients with accurate knowledge of outcome probabilities) ranged widely among the 17 studies, from 0.08 to 0.77. The slope of the univariate regression between logit control and ln(OR) in Figure 3, prior to bias correction (dotted line), was significant (slope = −0.436; *P* = 0.001); this relationship was slightly steeper (that is, strengthened) after bias correction (solid line, slope = −0.466). In the non-bias-corrected analysis, the control event rate accounted for just over half of the variability in effect size (*R*^2^ = 0.52).

The multiple regression, which combined IPDASi probabilities dimension score and control event rate, was significant (*P* = 0.007) and accounted for slightly more variability (*R*^2^ = 0.54). While effect modification due to control event rate was still significant in this model (*P* = 0.018), IPDASi probabilities dimension score lost significance (*P* = 0.561).

### GRADE assessment of evidence quality

The quality of the evidence supporting the use of decision aids for improving the accuracy of patient knowledge of outcome probabilities was assessed here as ‘moderate’ with the GRADE framework (Table 3). Disregarding any explanation of sources of heterogeneity provided in the current study, the same body of pooled evidence would be assessed as ‘low’ (GRADE table not shown) due to rating down for ‘inconsistency’.

**Table 3:** **GRADE**[20]**evidence quality assessment for the effect of decision aids on the accuracy of patient knowledge of outcome probabilities**

**Risk of bias**	**Inconsistency**	**Indirectness**	**Imprecision**	**Publication bias**	**Quality**
Serious^**a**^	No serious inconsistency^**b**^	No serious indirectness	No serious imprecision^**c**^	Unlikely^**d**^	⊕⊕⊕Ο MODERATE

a. Study-level risk of bias assessments reported in the 2011 Cochrane update were used, except for two newly extracted risk of bias items where outcome-level assessments were more appropriate (blinding and incomplete outcome data). No studies had a high risk of bias due to sequence generation (11 were unclear and 6 low), or allocation concealment (6 were unclear and 11 low). Only two studies representing a small weighting in the pooled analysis (Lerman *et al.*[28] and McAlister *et al*. [29]) had a high risk of bias due to incomplete outcome data (1 was unclear and 14 low). Ten studies could be considered to have a risk of bias due to inadequate blinding of outcome assessment (4 were evidently unclear and 3 were evidently low), but for these studies the accuracy of knowledge of outcome probabilities was generally assessed using objective *a priori* criteria, thus inadequate blinding of outcome assessment was not considered serious. Most studies of decision aids do not blind the personnel delivering the intervention, and risk of bias was rated down for this reason.

b. Inconsistency was not rated down since there was a uniform direction of effect with all studies favoring decision aids and a large proportion of the heterogeneity is explained by the variation in the control event rate.

c. Imprecision was not rated down since the confidence intervals for the pooled RR are uniformly greater than 1.25 (with greater relative effects predicted for lower control event rates), and this estimate is based on over 2,000 patients in each arm.

d. Investigation of reporting bias using funnel plots was not feasible for this outcome. Reporting bias was considered unlikely based on the thoroughness of the search and discussion provided in an earlier Cochrane update.

## Discussion

Our analysis of main effects of decision aids on the accuracy of patient knowledge of outcome probabilities includes unpublished data from three studies in addition to the 14 studies previously included in the 2011 Cochrane analysis for this outcome. Compared to this earlier analysis, the added data slightly increase the pooled relative risk (from 1.74 to 1.80) and maintain the finding that all studies uniformly favor decision aids; additionally, they slightly increase the level of heterogeneity (from *I*^2^ of 83% to 87%) [7]. As recognized in the previous Cochrane review [10], this substantial level of heterogeneity limits the interpretability of the random effects pooled estimate since it represents an average of possible real effects of decision aids that vary widely from setting to setting. Thus an investigation of the factors that may influence this variation is warranted to better understand the conditions under which decision aids have their greatest effects [2,3,5,6].

Given that factors underlying real variation of intervention effects can include study-level characteristics of the comparator or control intervention, the active intervention or the study population [2,3], the current investigation therefore analyzed the effects of three covariates chosen to represent each of these sources of variability. There was no evidence that the type of control intervention modifies either the effect size or the control event rate. This negative finding provides incidental support for an assumption integral to the third covariate analysis of effect modification by control event rate (see Methods). That is, any effect modification is unlikely to be confounded by the control intervention manipulating effect size via effects on the control event rate. Thus the control event rate can be more reliably interpreted as representing a study population’s baseline level of knowledge of outcome probabilities.

The second covariate, decision aid quality as represented by the IPDASi probabilities dimension score, was found to modify effect size, the positive relationship observed being consistent with the expectation that higher-quality decision aids produce larger effect sizes. Overall, this result provides tentative support for the predictive validity of the probabilities dimension of the IPDASi, although statistical significance is borderline (*P* = 0.037). Significance is lost, for example, when IPDASi probabilities dimension score is combined in multiple regression with control event rate – although for any bivariate regression to be sufficiently powered, a larger sample size would generally be advisable. Thus, additional studies are necessary to improve certainty regarding effect modification due to the IPDASi probabilities dimension score.

Nevertheless, there are reasons to expect that decision aid quality defined according to the IPDASi probabilities dimension does in reality modify the effectiveness of decision aids. Firstly, individual components of decision aid design on which the IPDASi probabilities dimension are based [14] are supported by a review of evidence providing biologic or theoretical plausibility [15]. Secondly, subgroup analysis in the 2011 Cochrane review provides direct evidence for at least one design feature – that using numbers rather than words in decision aids to communicate probabilities improves knowledge of those probabilities to a statistically significantly greater extent [7]. The components of decision aid design that may underlie variation in effect sizes are not restricted to the IPDASi probabilities domain, however, and the updated IPDAS review summarizing recent evidence for presenting probabilities [9] describes additional promising factors to explore in future analyses of effect modification. The effects of individual design factors were not examined here because of the top-down approach to selecting covariates and the decision to restrict their number to minimize the risk of detecting spurious effect modification due to multiple comparisons. The selection of specific factors for future analyses should consider both the theoretical plausibility of effect modification, and whether the selected design feature is likely to be consistently relevant for all decision aids since some features, such as those relevant only to screening decisions, restrict the sample size of studies available for analysis.

For the third covariate, control event rate, the negatively sloped relationship is highly significant (*P* = 0.001) and is slightly steeper after correcting for dependence of the effect measure on the control event rate, increasing confidence in true effect modification. Furthermore, when combined in multiple regression with IPDASi probabilities dimension scores added to the model, control event rate remained significant despite the low power for this bivariate analysis. In univariate analysis, approximately half the heterogeneity is accounted for by the control event rate. Thus control event rate, reflecting patients’ baseline level of knowledge of outcome probabilities, appears to be an important variable explaining heterogeneity of effects of decision aids on accuracy of knowledge of outcome probabilities, with greater relative effects expected when the baseline proportion of patients with accurate knowledge is lower.

**Figure 4:**
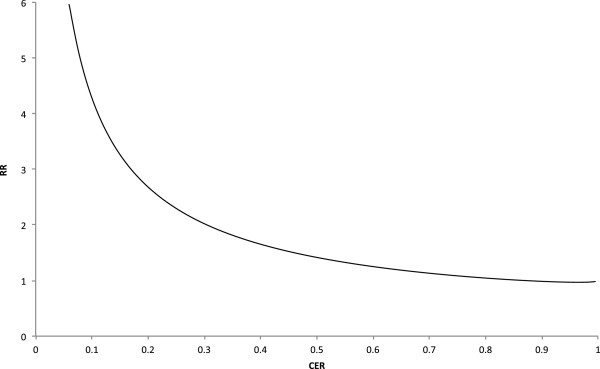
**Empirically fitted relationship predicting relative risk when the control event rate (baseline knowledge) is known.** CER, control event rate; RR, relative risk.

The precise relationship between control event rate and effect size is not intuitive from the meta-regression in Figure 3, since both variables are on the logarithmic scale. To facilitate interpretation, the relationship was back-translated to show how the effect sizes are expected to vary over a range of control event rates, using relative risk, the effect measure commonly reported in the literature. The relationship thus represented in Figure 4 could have various predictive uses, such as for planning future trials evaluating decision aids. For example, when a control event rate of 0.5 is anticipated based on pilot work, the corresponding expected relative risk (of 1.4) could inform decisions about proceeding with the full trial.

Given the clinical utility of being able to define what types of patients benefit most from an intervention using the relationship between effect size and the control event rate (or level of baseline risk), one may ask how often is such a relationship significant for interventions in other contexts, and why is it not characterized more frequently? An analysis by Schmid and colleagues provides an informative answer [5]. They examined 115 meta-analyses of clinical trials to detect whether there was an effect of control event rate on effect size. After correcting for dependence of the effect measure on control event rate and using a two-standard error rule of significance, they found linear correlations with ln(OR) in only 14% of meta-analyses. They proposed that such effect modification is more likely to be found when the meta-analysis includes a sufficient number of studies (ten or more), and comprises greater variation in control event rates across included studies. The current meta-analysis, which includes 17 studies and has widely ranging control event rates (0.08 to 0.77), is consistent with this observation. This follows from the idea that ‘heterogeneity is your friend’ since more heterogeneity provides a better opportunity to detect a covariate effect [38,39].

Finally, by providing an explanation for heterogeneity, the quality of the pooled research was assessed with the GRADE framework [19,20] as ‘moderate’ instead of ‘low’. This reflects that the current investigation of sources of heterogeneity improves the quality of the evidence from a body of 17 pooled studies by improving its interpretability and utility [2-4].

A limitation of investigating study-level sources of heterogeneity is that interpretation may be affected by confounding from other study-level factors, particularly those related to study design [2]. These factors include both methodological aspects known to increase the risk of bias in an RCT (sequence generation, allocation concealment, blinding of patients, blinding of intervention providers, blinding of outcome assessors, and completeness of outcome data) and aspects of outcome measurement [2]. Confounding by aspects of outcome measurement requires considering characteristics of the questions used to measure knowledge of probabilities. Evaluation questions can vary, for example, in the number of categories to select between within a multiple choice question, whether the question forces guessing (by not providing an option for ‘unsure’), whether numbers or words are used to represent probabilities, and in the precision used to define the probability ranges for each category. Some but not all of these characteristics are also design features of decision aids whose influence on improving knowledge of probabilities has been established – for example, that presenting probabilities as numbers is more effective than words [7,40,41]. Similarly, specific characteristics would be expected to influence the difficulty of evaluation questions. Although there is extensive research and standards that guide and support the presentation of probabilities in decision aids [14,15], research into how relevant characteristics affect the difficulty of evaluation questions – and therefore influence the measurement of patient knowledge of probabilities – is lacking. It was not possible to conduct an analysis of these effects in the current study since descriptions of evaluation questions were not available for most studies. Considering how question difficulty has the potential to influence and confound estimates of baseline knowledge (control event rates), future research into this measurement issue is warranted.

## Conclusions

The current sub-analysis increases the interpretability and utility of previously pooled evidence on the effects of decision aids for improving accuracy of knowledge of outcome probabilities by adding data for this outcome and characterizing the effects of two potential contributors to heterogeneity of decision aid effects: decision aid quality and the control event rate. While decision aid quality, as measured by the IPDASi probabilities dimension, may increase the effects of decision aids, this finding is of borderline significance and requires further analysis with data from additional studies. The control event rate – representing patients’ baseline level of knowledge of outcome probabilities – is a highly significant and substantial contributor to heterogeneity, with greater relative effects observed when the baseline proportion of patients with accurate knowledge is low. This suggests that decision aids are most effective in populations with low awareness. Further research may be warranted, however, to determine whether aspects of evaluation questions influence the measurement of knowledge of probabilities. Knowledge of how relative risk is expected to vary across a wide range of control event rates may be useful to inform policy judgments about the uptake of decision aids to inform patients of probabilities related to the outcomes of interventions or diagnostic tests in specific settings.

## Abbreviations

CER: Control event rate; df: Degrees of freedom; IPDASi: International patient decision aid standards instrument; OR: Odds ratio; RCT: Randomized controlled trial; RR: Relative risk; WLS: Weighted least squares.

## Competing interests

The authors declare that they have no competing interests. There was no funding for this study.

## Authors’ contributions

SG designed the study, obtained unpublished data, conducted the analysis, and drafted and revised the manuscript. DS and CB provided guidance during the conception of the study and contributed critical revisions. MA extracted study data as a second reviewer. SW provided statistical guidance and contributed critical revisions. All authors read and approved the final manuscript.
